# Effects of Alcohol on the Cardiovascular System in Women

**DOI:** 10.35946/arcr.v40.2.12

**Published:** 2020-08-06

**Authors:** Mariann R. Piano, Laurel A. Thur, Chueh-Lung Hwang, Shane A. Phillips

**Affiliations:** 1 School of Nursing, Vanderbilt University, Nashville, Tennessee; 2 Department of Physical Therapy, University of Illinois at Chicago, Chicago, Illinois

**Keywords:** alcohol, cardiovascular disease, hypertension, stroke, women

## Abstract

Cardiovascular disease (CVD) is a leading cause of morbidity and mortality for women. This review summarizes the relationship between alcohol consumption and common CVDs in women and highlights potential differences from men. Except for risk of hypertension, no sex-related effects of alcohol consumption on the risk for coronary heart disease and stroke have been reported, and data on the sex-related effects on risk for peripheral arterial disease are limited. For women, alcohol consumption has a J-shaped relationship with hypertension. About 1 to 2 standard drinks per day is associated with lower risk for the development of hypertension, whereas for men, the relationship is relatively linear. In the area of alcoholic cardiomyopathy, the prevalence is greater for men, but women may develop alcoholic cardiomyopathy at a lower lifetime level of alcohol consumption. Overall, data support that 1 to 2 standard drinks per day for women and men is associated with a lower risk of CVD, and higher daily amounts may increase the risk of CVD.

## INTRODUCTION

Biologic sex is an important determinant of health and disease. Over the past several decades, research has revealed sex differences in the epidemiology, risk, clinical manifestations, pathophysiology, and progression of many diseases, including cardiovascular disease (CVD) and alcohol-induced pathologies. CVD is the leading cause of morbidity and mortality for women.^1^ Therefore, understanding the potential interaction between sex and alcohol consumption on the cardiovascular system is important. This review presents the effects of alcohol consumption on the cardiovascular system in women, focusing on prevalent cardiovascular conditions such as hypertension, coronary heart disease (myocardial infarction), stroke, and peripheral arterial disease. The cardiovascular disorders are briefly defined, sex differences in the prevalence and prognosis of the disorders are discussed, followed by examination of the sex differences in alcohol’s effects on those conditions. This review also reports on the sex differences in the prevalence, clinical manifestations, and outcomes associated with alcoholic cardiomyopathy.

## METHODS

The relationship between alcohol consumption and CVD has been extensively investigated, and women have been included in many of these studies. However, results for women and men often have not been presented separately. Studies have been experimental, such as short-term clinical trials, and longitudinal, using participants from ongoing population cohorts such as the Nurses’ Health Study and the Framingham Heart Study. Data from these studies have allowed for the completion of several comprehensive systematic reviews and meta-analyses of alcohol use and its relationships with hypertension, myocardial infarction, stroke, and peripheral arterial disease.

This review summarizes data from meta-analyses and from longitudinal studies. Results were included in this discussion if they were reported for men and women separately. Data were summarized from studies that examined alcohol consumption by either standard drinks or by grams per unit of ethanol per day or week. Studies varied in the way they measured and categorized alcohol consumption, making comparisons sometimes challenging. Most studies reviewed reported alcohol consumption in grams of ethanol per day or week and defined a standard drink as 12 to 15 grams of alcohol.

Drinking patterns, particularly binge drinking, have emerged as an important modifier of the relationship between alcohol and cardiovascular risk. For a review of the sex differences in binge drinking on cardiovascular function, see the 2017 review by Piano and colleagues.^2^

## BLOOD PRESSURE AND HYPERTENSION

The current blood pressure guidelines published in 2018 define hypertension as systolic blood pressure (SBP) greater than 130 mm Hg or diastolic blood pressure (DBP) greater than 80 mm Hg.^3^ In many of the studies reviewed for this article, hypertension was defined as SBP greater than 140 mm Hg or DBP greater than 90 mm Hg. The effects of alcohol consumption on blood pressure in women are important to consider, because hypertension is a leading cause of cardiovascular morbidity and mortality.^1^ From 2015 to 2016, the overall prevalence of hypertension (SBP > 140 mm Hg) among adults was 29.0% and was similar for men (30.2%) and women (27.7%).^4^ For adults ages 18 to 59, hypertension prevalence is greater for men than for women. However, women older than age 60 have greater prevalence (66.8%) of hypertension than men older than age 60 (58.5%). In the United States, experts have estimated that alcohol consumption accounts for 10% of the population burden of hypertension.^3^

The effects of single episodes of alcohol consumption have not been investigated in women. Studies that included men have reported that consuming alcohol (1 to 2 standard drinks) in a single episode was associated with transient blood pressure increases that ranged from 4 to 7 mm Hg SBP and from 4 to 6 mm Hg DBP.^5^–^7^ In healthy women, an episode of low to moderate alcohol consumption (1 to 2 standard drinks, or 12 to 14 grams of ethanol) more than likely would have no appreciable effect on blood pressure.

Clinical studies and randomized clinical trials have been designed to examine the short-term effects of alcohol consumption, but only one study included women.^8^–^12^ In a crossover study, Mori and colleagues examined the effects of different levels of alcohol consumption on ambulatory, 24-hour blood pressure levels among healthy, premenopausal women ages 20 to 45 (*N* = 24).^12^ Blood pressure was measured after a 4-week period during which participants consumed different amounts of red wine: 42 to 73 grams of alcohol per week (0.5 to 1.0 drink per day) vs. 146 to 218 grams of alcohol per week (2 to 3 drinks per day). Awake SBP was 2.3 mm Hg higher and DBP was 1.3 mm Hg higher for women who consumed more alcohol (2 to 3 drinks per day) than for women who consumed less (0.5 to 1.0 drink per day) or no alcohol. For the women who consumed less, the red wine showed no effect on blood pressure. These findings suggest 2 to 3 drinks per day have a mild pressor effect on blood pressure in women.

Findings from two meta-analyses support an association between alcohol consumption and the risk of developing hypertension (SBP > 140 mm Hg), and that sex is a modifier of this relationship.^13^,^14^ In a meta-analysis of 18 cohort studies, Roerecke and colleagues found that for women (with a mean age of 46.7), compared to abstainers, consumption of 1 to 2 drinks per day was not associated with increased hypertension risk.^14^ However, hypertension risk was elevated for women who consumed 3 or more drinks per day, with a relative risk (RR) of 1.42, 95% confidence interval (CI) [1.22, 1.66]. However, for men, compared to abstainers, any level of alcohol consumption increased the risk for hypertension.

In another meta-analysis conducted several years earlier, Briasoulis and colleagues reported that women ages 30 to 55 who consumed less than 10 grams of alcohol (less than 1 drink) per day, compared to abstainers, showed a significant reduction in RR for hypertension (RR = 0.87, 95% CI [0.82, 0.92]).^13^ These investigators also reported a “trend toward decreased risk of hypertension” for women participants who consumed 1 to 2 drinks per day (RR = 0.9, 95% CI [0.87, 1.04]). For women, the increased risk for hypertension emerged at alcohol consumption levels of 21 to 30 grams (2 drinks) per day and 31 to 40 grams (2 to 3 drinks) per day. Men who consumed less alcohol showed a trend toward increased risk for hypertension, and a significant increase in risk of hypertension was shown among men who consumed 31 to 40 grams per day (RR = 1.77, CI [1.39, 2.26]) or more than 50 grams (about 4 drinks) per day (RR = 1.62, CI [1.31, 1.87]). Results from another meta-analysis also found a linear relationship for men and a J-shaped relationship for women.^15^

Roerecke and colleagues also conducted a meta-analysis to examine the effects of a reduction in alcohol consumption on blood pressure.^16^ Data were analyzed from 36 clinical trials, and the main analysis included men (*n* = 2,464) and women (*n* = 401) together, with a subgroup analysis for sex differences. Although trial characteristics (e.g., length of trial and blood pressure assessment method) differed among the studies, for individuals who drank more than 2 drinks per day, a reduction in alcohol consumption was associated with a reduction in blood pressure. Blood pressure reductions were greatest for individuals who consumed 6 or more drinks per day at baseline.

These same authors estimated the reduction in blood pressure that might be achieved by 50% of people in the United Kingdom who consume more than 2 drinks per day.^16^ Using pooled effect sizes from a subgroup analyses for sex and amount of alcohol, the authors estimated the proportional difference, or the magnitude of SBP reduction, would be a 4.4% reduction for men and a 1.2% reduction for women among men and women with SBP greater than 140 mm Hg.

These results and the examination of daily alcohol consumption indicate the relationship between alcohol and blood pressure is different for men (linear) and for women (J-shaped). For men, all levels of alcohol consumption are associated with increased blood pressure and risk of hypertension.^14^ For women, the J-shaped relationship indicates that 1 to 2 drinks per day has no effect^14^ or a lowering effect^13^ on blood pressure, whereas more than 2 drinks per day increases the risk of hypertension. All these meta-analyses included women before and after menopause; therefore, speculation about the potential effect of hormones on these sex differences is difficult.

## CORONARY HEART DISEASE

Coronary heart disease is defined as a disease that results from coronary artery disease or myocardial infarction. The terms “coronary heart disease” and “coronary artery disease” are often used interchangeably. Across all age groups, the prevalence of coronary heart disease and myocardial infarction is greater for men than for women.^1^ However, the difference in prevalence between men and women narrows with advancing age.^1^,^17^ Most women are older when they present with their first myocardial infarction (the mean age is 71.8). Regardless of age, more women than men die within 1 to 5 years after a first myocardial infarction.^17^

Many epidemiologic studies have examined the relationship between coronary heart disease (and myocardial infarction) and alcohol use. Ronksley and colleagues have conducted the most comprehensive meta-analysis, which incorporated 84 studies over the past 30 years.^18^ Among those studies, 52% included women. In the analysis, active drinkers were defined using a wide range of alcohol consumption categories, from less than 2.5 grams per day (less than 0.5 drink) to more than 60 grams per day (5 or more drinks). For comparison, the reference group was nondrinkers. These investigators reported that for men and women ages 15 to 90, any amount of alcohol consumption compared to none was associated with a reduced RR for occurrence of coronary heart disease and mortality (for men: RR = 0.71, 95% CI [0.66, 0.77] and RR = 0.77, 95% CI [0.72,0.82], respectively; for women: RR = 0.71, 95% CI [0.66, 0.77] and RR= 0.78, 95% CI [0.64, 0.94], respectively).

In an analysis that used data from the international Interheart case-control study, which included a population of women and men with a mean age of 58, Leong and colleagues examined the relationship between alcohol consumption of 1 drink or more per day during the year before a myocardial infarction.^19^ Women who had at least 1 drink were less likely to have a myocardial infarction (*OR* = 0.73, 95% CI [0.61, 0.88]) compared to men who had at least 1 drink (*OR* = 0.92, 95% CI [0.84, 1.00]). Because alcohol use was defined as the consumption of any alcoholic beverage within the previous 12 months, this analysis did not allow for determining any dose response or specific level of alcohol consumption associated with the reduced risk in women or men.

Collectively, based on these data, there are no sex-related effects of alcohol consumption on the risk for occurrence of coronary heart disease or for coronary heart disease mortality. The results reported by Ronksley and colleagues suggest a wide range of daily alcohol consumption levels are associated with a reduced risk of incidence of coronary heart disease and coronary heart disease mortality in women and men.^18^ The lack of specific alcohol intake categories in the Interheart study disallows understanding either the lower or upper limits for alcohol consumption associated with myocardial infarction risk.

Neither study’s findings provide specific guidance for understanding the frequency or regularity of consuming different daily levels of alcohol within a designated time period, for example, per week or month. More than likely, more than 2 to 3 drinks per day, every day, may be associated with a different risk profile when compared to consuming 2 to 3 drinks per day, but only 2 to 3 times per week. Finally, consuming 5 or more drinks per day could be considered a binge pattern, which is associated with increased risk of CVD.^2^

## STROKE

The two main types of stroke are ischemic and hemorrhagic. Both types are associated with a marked reduction in cerebral blood and oxygenation and involve ischemic cell death. Approximately 90% of strokes are ischemic and arise from a decrease or blockage of cerebral blood flow, whereas about 10% of strokes are due to intracerebral hemorrhage.^1^ Stroke is the most common cerebrovascular disease and the second-leading cause of death worldwide. Each year, approximately 55,000 more women than men have a stroke. For women younger than age 75, stroke incidence rates are lower than they are for men, although for women older than age 75, incidence rates exceed those for men older than age 75. A similar age-related trend is found for stroke-associated mortality rates, which are similar for women and men younger than age 45. Women between ages 45 and 74 have lower stroke mortality than men, but as age advances, mortality rates tend to be higher for women compared to age-matched men.

Among a Swedish cohort of men and women, Larsson and colleagues examined the association between alcohol consumption and risk of different stroke subtypes.^20^ The reference group included nondrinkers, never drinkers, and occasional drinkers. Among women and men, no statistically significant association was found between any level of alcohol consumption (i.e., less than 1 to more than 21 drinks per week) and the risk of ischemic stroke. For men and women, only the higher level of alcohol consumption (more than 21 drinks per week) was associated with increased risk of intracerebral hemorrhagic stroke. For women but not men, all levels of alcohol consumption were significantly and positively associated with subarachnoid hemorrhagic stroke. As the authors noted, at the highest level of alcohol consumption and among this cohort of women, the number of cases of all stroke subtypes (*n* = 2 to *n* = 11) was low, which affects the power of these associations.

In this same study, a meta-analysis was conducted and included 27 prospective studies, of which the majority controlled for potential confounders such as age, sex, smoking, body mass index, and diabetes mellitus.^20^ In the subgroup analysis, 2 drinks or fewer per day for women was associated with a lower risk for ischemic stroke (RR = 0.80, 95% CI [0.83, 0.95]). No effect was shown for intracerebral hemorrhagic stroke (RR = 0.95, 95% CI [0.76, 1.19]), and a modest increase was shown for subarachnoid hemorrhagic stroke (RR = 1.38, 95% CI [1.01, 1.85]). More than 2 drinks per day for women was associated with increased RR for all stroke subtypes. Similar findings were reported for men. For women compared to men, more than 2 drinks per day appeared to be a greater RR for intracerebral and subarachnoid hemorrhagic stroke, but no sex differences were found in the RR values for all stroke subtypes.

Zheng and colleagues conducted a meta-analysis of prospective observational studies (23 studies including 18 cohorts) and examined the association of alcohol intake and the risk of cardiovascular outcomes, which included total stroke (ischemic and all hemorrhagic strokes) and ischemic stroke.^21^ For men and women, less than 15 grams per day of alcohol had no effect on the risk for total stroke, whereas for both men and women, this low daily amount of alcohol consumption had similar significant reductions in the risk for ischemic stroke (about 17% to 24%). For women but not for men, moderate alcohol consumption (15 to 30 grams per day) was associated with a reduction of risk for total stroke and ischemic stroke. However, the RR ratio (women to men) was not significantly different, indicating no sex difference. For men and women, heavy alcohol consumption had no significant effect on total or ischemic stroke risk.

Jimenez and colleagues examined the relationship of alcohol and the risk of stroke for women enrolled in the prospective Nurses’ Health Study.^22^ Women (with a mean age of 46) free of CVD at baseline were followed between 1980 and 2006. Women who reported light (up to 4.9 grams per day) to moderate (5.0 to 14.8 grams per day) alcohol consumption had a lower risk of stroke compared to abstainers. Consumption of 30 to 45 grams per day had no effect on stroke risk, whereas consumption exceeding 36 grams per day (about 3 drinks) was associated with greater risk of stroke. Similar results were obtained after a multivariate analysis controlled for many key variables, such as age, aspirin use, hormone replacement therapy, and smoking. However, as the authors noted, the confidence limits were wide, because only a few events were at the higher end of the alcohol intake range. Few women in this study consumed more than 45 grams per day, limiting the power to investigate that level of alcohol consumption on stroke risk.

Collectively, based on the data reviewed, there are no sex-related differences of alcohol consumption on the risk for total stroke or stroke subtypes. Findings from these studies suggest a J-shaped relationship for alcohol use and stroke risk for both men and women, with 1 to 2 drinks being not harmful for women and perhaps reducing the risk for certain stroke subtypes.^20^,^21^ Reported findings for higher levels of alcohol consumption are similar for men and women; that is, exceeding 3 drinks per day or 21 drinks per week may increase the risk of all types of stroke, particularly hemorrhagic stroke.

## PERIPHERAL ARTERIAL DISEASE

Peripheral arterial disease is an atherosclerotic, occlusive disease of the lower limbs affecting 202 million individuals worldwide.^1^ Unlike the other CVDs discussed in this article, less is known about peripheral arterial disease in women compared to men.^23^ Many population-based prevalence studies of peripheral arterial disease have not noted prevalence separately for women. Overall, the disease progression, pathophysiology, and symptoms in women have been poorly characterized. For men and women, prevalence of peripheral arterial disease increases with age, reaching 25% for women older than age 80. Investigators often use the ankle brachial index (ABI), which is calculated by dividing the SBP at the ankle by the SBP at the arm, as a metric for diagnosing peripheral arterial disease. Hirsch and colleagues used the ABI test and reported that the severity of peripheral arterial disease for women was similar to the severity associated with men.^23^

Compared to the relationships between alcohol consumption and coronary heart disease or stroke, the relationship between alcohol consumption and peripheral arterial disease has been examined less often. Consequently, no meta-analyses or systematic reviews have been reported. More than a decade ago, two large prospective studies examined the effects of alcohol consumption on peripheral arterial disease.^24^,^25^ The Strong Heart Study was conducted in the United States, and the Rotterdam study was done in Europe, and both used an ABI value of less than 0.9 as an indicator for the presence of peripheral arterial disease.

In the Strong Heart Study, which enrolled only American Indian participants, current alcohol drinking was inversely associated with peripheral arterial disease prevalence for men and women, after controlling for other factors.^24^ Because more specific information was not provided about levels or amounts of alcohol consumption and only American Indians were included, the generalizability of the findings is limited.

In the Rotterdam study, however, results were similar.^25^ This study enrolled 1,489 men and 2,486 women who were age 55 or older and were free from CVD at baseline. These studies found that the risk of peripheral arterial disease (assessed by an ABI of less than 0.9) was significantly reduced (22% to 36%) for women who reported consuming 10 to 20 grams of ethanol, or less than 2 drinks, per day (*OR* = 0.66, 95% CI [0.43, 1.00]) or more than 20 grams per day (*OR* = 0.64; 95% CI [0.41, 1.01]). A nonsignificant association was found for men. However, risk was not lowered for women smokers, men smokers, or men nonsmokers, suggesting that alcohol consumption may lower risk of peripheral arterial disease for women who do not smoke.

These findings suggest there are sex-related effects of alcohol consumption on the risk for peripheral arterial disease.^24^,^25^ For women, unlike for men, low to moderate drinking levels may reduce the risk of peripheral arterial disease. Many studies have examined alcohol and the other CVDs, but a dearth of studies have examined alcohol and peripheral arterial disease. Therefore, more research on the effect of alcohol consumption on peripheral arterial disease in women is warranted.

## ALCOHOLIC CARDIOMYOPATHY

The term “alcoholic cardiomyopathy” describes a heart muscle disease found in individuals with a history of heavy, long-term, alcohol consumption. Alcoholic cardiomyopathy is characterized by a dilated left ventricle, normal or reduced left ventricle wall thickness, increased left ventricle mass, and (in advanced stages) a reduced left ventricle ejection fraction (less than 40%).^26^ In studies of alcoholic cardiomyopathy, women have been excluded or underrepresented. In part, this may relate to differences and overall lower alcohol consumption and prevalence of alcohol use disorders in women compared to men.

At a population level, the exact prevalence of alcoholic cardiomyopathy, especially among women, remains unknown. Two decades ago, cross-sectional studies that estimated the frequency of alcoholic cardiomyopathy among individuals with a diagnosis of idiopathic dilated cardiomyopathy generally excluded women.^27^–^29^ Using a large, nationally representative database of inpatients, Mogos and colleagues recently estimated that the prevalence of new or existing alcoholic cardiomyopathy was 68 per 100,000, or 1 for every 1,471 inpatient hospitalizations.^30^ Among this inpatient population, a greater percentage of men than women had alcoholic cardiomyopathy (the male to female ratio was 8 to 1).

Some reports indicated that women with alcohol dependence developed alcoholic cardiomyopathy after consuming less alcohol over a shorter period than age-matched men with alcohol dependence.^31^ However, for women and men, the exact amount and duration of alcohol consumption associated with the development of alcoholic cardiomyopathy remains unknown. Also, the point at which alcohol-induced abnormalities appear during the course of an individual’s lifetime of drinking is not well-established and is highly individualized, suggesting either protective or adverse interaction effects because of genetic or lifestyle modifications.^26^

Urbano-Márquez and colleagues prospectively enrolled a cohort of women with alcoholic cardiomyopathy and specifically examined myocardial structural characteristics (e.g., ventricular dimensions and mass).^32^ In this study, asymptomatic women (*n* = 50) and men (*n* = 100) with alcoholic cardiomyopathy had similar changes in echocardiographic parameters reflecting myocardial function and structure, suggesting similar alcohol-induced, global myocardial changes. Women had a total lifetime dose of ethanol (14.2 ± 5.4 g/kg of body weight) that was less than the dose for men (23.1 ± 12.4 g/kg of body weight), leading to the idea that women may be more vulnerable to the development of alcoholic cardiomyopathy.

Recently, Mogos and colleagues examined sex differences in the distribution of co-occurring conditions among men and women with alcoholic cardiomyopathy.^30^ Women with alcoholic cardiomyopathy, compared to men with the same condition, were significantly more likely to have co-occurring anemia (28.3% vs. 19.2%), heart failure with preserved ejection fraction (3.2% vs. 2.5%), thyroid disorders (10.5% vs. 4.7%), and asthma (5.1% vs. 2.5%). Women were also more likely than men to experience co-occurring alcoholic cardiomyopathy and depression or anxiety. Conversely, men with alcoholic cardiomyopathy were more likely than women to also have hyperlipidemia (19.5% vs. 15.5%), heart failure with reduced ejection fraction (18.6% vs. 15.2%), diabetes mellitus (18.0% vs. 13.6%), and renal disease (21.8% vs. 18.4%).

Findings from the study suggest sex differences in certain co-occurring conditions among men and women with alcoholic cardiomyopathy.^30^ Prevalence of alcoholic cardiomyopathy is greater for men compared to women, and women may develop the condition after shorter lifetime alcohol consumption. Women with alcoholic cardiomyopathy experienced more anxiety and depression; however, these findings are similar to those found in the general population.^33^ Nonetheless, co-occurring conditions are important considerations when treating women who have alcoholic cardiomyopathy.

## CONCLUSION

Many studies have included women in the examination of the effects of alcohol on the cardiovascular system. Excluding alcoholic cardiomyopathy and other alcohol-induced diseases such as liver disease,^34^ low to moderate alcohol consumption does not adversely affect cardiovascular risk in women. Although consumption of 2 to 3 drinks per day may exert a pressor effect on blood pressure in women,^12^ low to moderate levels of daily alcohol consumption (1 to 2 drinks per day) have been associated with no increased risk of hypertension.^14^

Furthermore, with the exception of hypertension, sex-specific differences have not been found in the relationship between alcohol and cardiovascular risk. Results from these studies have been informative, but a lack of data remains for understanding the safe or risk-reducing weekly limits of alcohol consumption for women.

Results from some of the studies pose a bit of conundrum. Zheng and colleagues reported that moderate levels of alcohol consumption in women were associated with a lower risk for total stroke and ischemic stroke, but this same level of alcohol for women, compared to men, has been reported to have a significant 10% increase in the risk of total mortality.^21^

What might the recommended levels of alcohol consumption for women be? The answer lies in the recent, large-scale, international study by Wood and colleagues.^35^ These investigators analyzed individual participant data from three large, international sources (including about 600,000 current drinkers). These investigators found the threshold for the lowest risk for all-cause mortality (i.e., mortality related to any condition or event) for men and women was about 100 grams of alcohol (about 7 drinks) per week. Furthermore, women who reported drinking more than the U.K. or U.S. recommended weekly limits of 112 grams per week^36^ and 98 grams per week,^37^ respectively, had a shorter life expectancy (by about 1.1 to 1.5 years) at age 40, compared with women who reported drinking less than these thresholds.^35^

Alcohol consumption remains a major risk factor for global burden of disease.^38^ For women who consume alcoholic beverages, the Dietary Guidelines for Americans recommend drinking in moderation—up to one drink per day.^37^ The Guidelines also recommend that individuals who do not drink alcohol not start drinking for any reason.
